# Dorsal root ganglion-derived exosomes deteriorate neuropathic pain by activating microglia via the microRNA-16-5p/HECTD1/HSP90 axis

**DOI:** 10.1186/s40659-024-00513-1

**Published:** 2024-05-15

**Authors:** Yinghao Xing, Pei Li, Yuanyuan Jia, Kexin Zhang, Ming Liu, Jingjing Jiang

**Affiliations:** Department of Anesthesiology, Shengjing Hospital of China Medical University, No. 36, Sanhao Street, Heping District, Shenyang, 110004 Liaoning People’s Republic of China

**Keywords:** Neuropathic pain, microRNA-16-5p, HECTD1, HSP90, Dorsal root ganglion-derived exosomes

## Abstract

**Background:**

The activated microglia have been reported as pillar factors in neuropathic pain (NP) pathology, but the molecules driving pain-inducible microglial activation require further exploration. In this study, we investigated the effect of dorsal root ganglion (DRG)-derived exosomes (Exo) on microglial activation and the related mechanism.

**Methods:**

A mouse model of NP was generated by spinal nerve ligation (SNL), and DRG-derived Exo were extracted. The effects of DRG-Exo on NP and microglial activation in SNL mice were evaluated using behavioral tests, HE staining, immunofluorescence, and western blot. Next, the differentially enriched microRNAs (miRNAs) in DRG-Exo-treated microglia were analyzed using microarrays. RT-qPCR, RNA pull-down, dual-luciferase reporter assay, and immunofluorescence were conducted to verify the binding relation between miR-16-5p and HECTD1. Finally, the effects of ubiquitination modification of HSP90 by HECTD1 on NP progression and microglial activation were investigated by Co-IP, western blot, immunofluorescence assays, and rescue experiments.

**Results:**

DRG-Exo aggravated NP resulting from SNL in mice, promoted the activation of microglia in DRG, and increased neuroinflammation. miR-16-5p knockdown in DRG-Exo alleviated the stimulating effects of DRG-Exo on NP and microglial activation. DRG-Exo regulated the ubiquitination of HSP90 through the interaction between miR-16-5p and HECTD1. Ubiquitination alteration of HSP90 was involved in microglial activation during NP.

**Conclusions:**

miR-16-5p shuttled by DRG-Exo regulated the ubiquitination of HSP90 by interacting with HECTD1, thereby contributing to the microglial activation in NP.

## Background

Neuropathic pain (NP) is a term for a series of conditions induced by a lesion or disease of the parts of the nervous system, and allodynia (pain results from a stimulus that does not usually provoke pain) and hyperalgesia (increased pain due to a stimulus that usually provokes pain) are prominent symptoms [1]. Microglia, a kind of immune cell population in the central nervous system (CNS), has attracted wide attention in rodent models of NP that develop strong mechanical and thermal hypersensitivity, and microglial activation is observed in the dorsal horn of the spinal cord [2]. Under normal conditions (when dysfunction of neurons or other cell types is not pre-existing), stimulating microglia induces pain hypersensitivity, highlighting their capacity to cause pain [3, 4]. Inhibiting the function of microglia suppresses the aberrant excitability of dorsal horn neurons and NP, underlining its potential target for treating NP [5].

Exosomes (Exo), a subtype of extracellular vesicles implicated in intercellular communication with the capability to transfer cargoes, including protein, lipids, and nucleic acids, have been the focus of many pain states [6]. For instance, extracellular vesicle-encapsulated microRNA (miR)-23a from dorsal root ganglia (DRG) neurons have been reported to promote macrophage polarization following peripheral nerve injury [7]. However, there are no clues about whether DRG-Exo can mediate microglial activation in NP. Although Exo can release the contents directly into the cytoplasm by fusing with the outer membrane of the recipient cell, endocytosis of Exo by the recipient cell is the most common uptake mechanism [8]. Considering the importance of miRNAs in neuroimmune communication in the DRG in preclinical chronic pain [9], and the long history of our team studying miRNAs in the CNS [10, 11], we screened out a miRNA delivered by DRG-Exo regulating the microglial activation in NP. Blasi’s research group modified the successfully immortalizing murine macrophages to generate the BV2 microglial cells expressing 90% positive for microglia cell markers [12], which were used as a cell model in this study. By the activation of the microglial BV2 cell line with lipopolysaccharide (LPS) [13, 14] and the application of DRG-Exo, we identified miR-16-5p as one of the most upregulated miRNAs in BV2 cells upon DRG-Exo co-culture. The pro-apoptotic effect of sarcopenia-derived miR-16-5p has been demonstrated as a contributor to the exacerbation of myocardial infarction [15]. More relevantly, mmu-miR-16-5p has been predicted to be associated with the pathogenetic process of NP [16] whose role has not been clarified yet. Therefore, we examined the effects of DRG-Exo on spinal nerve ligation (SNL)-induced NP in mice in the present study and investigated whether miR-16-5p delivered by DRG-Exo was involved in this process and the detailed mechanism.

## Results

### DRG-Exo are involved in NP in mice induced by SNL

**Fig. 1:**
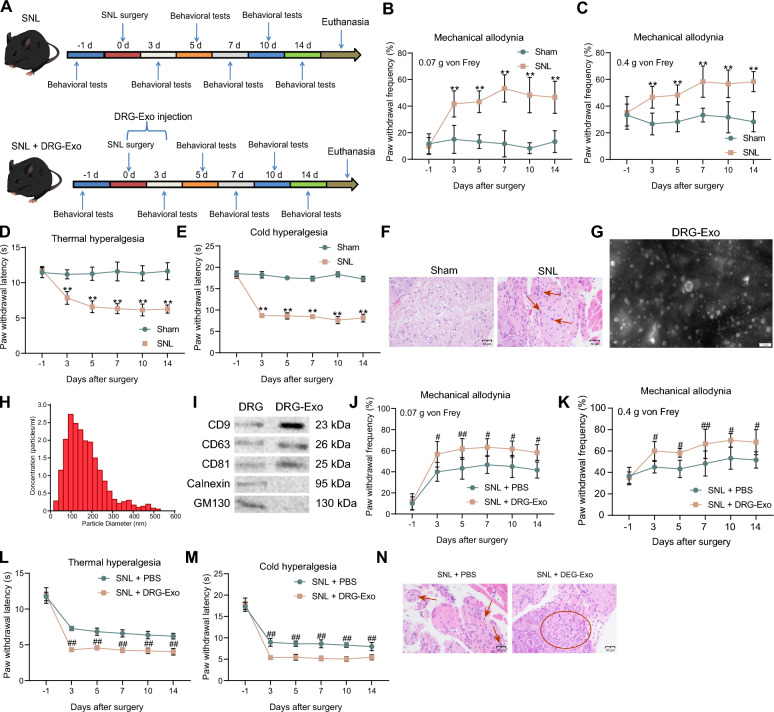
DRG-Exo are involved in NP in SNL-induced mice. **a** Flow chart of mouse SNL modeling and DRG-Exo injection. Assessment of mechanical allodynia response in SNL mice using 0.07 g (**b**) or 0.4 g (**c**) von Frey filaments at different time points before and after SNL surgery, respectively. **d** The responses of SNL mice to thermal hyperalgesia were evaluated at different time points before and after SNL surgery. **e** The responses of SNL mice to cold hyperalgesia were assessed at different time points before and after SNL surgery, respectively. **f** Inflammatory infiltration in the spinal cord (L4) of SNL mice was observed using HE staining. **g** The morphology of DRG-Exo was observed using TEM. **h** The diameter of DRG-Exo was analyzed using NTA. **i** Surface markers CD9, CD63, CD81 expression and contaminant proteins Calnexin and GM130 in DRG-Exo using western blot. Assessment of mechanical allodynia response in PBS- or DRG-Exo-treated mice using 0.07 g (**j**) or 0.4 g (**k**) von Frey filaments at different time points before and after SNL surgery, respectively. **l** The responses of PBS- or DRG-Exo-treated mice to thermal hyperalgesia were evaluated at different time points before and after SNL surgery. **m** The responses of PBS- or DRG-Exo-treated mice to cold hyperalgesia were assessed at different time points before and after SNL surgery, respectively. **n** Inflammatory infiltration in the spinal cord (L4) of PBS- or DRG-Exo-treated mice observed using HE staining (arrows represent immune cells and the circle represents the enrichment of immune cells). Values are expressed as mean ± SD. **p < 0.01 vs the sham group and #*p* < 0.05, ##*p* < 0.01 vs the SNL + PBS group by two-way ANOVA

A mouse model of NP was developed by SNL, and the mice were subjected to behavioral assessment of mechanical allodynia, thermal hyperalgesia, and cold hyperalgesia on the first day before the surgery and at the 3rd, 5th, 7th, 10th, and 14th d postoperatively (Fig. 1A). Mechanical allodynia showed that SNL-treated mice had prolonged mechanical abnormalities in pain, with a significant increase in the frequency of paw withdrawal stimulated by up to 0.07 g and 0.4 g von Frey filaments (Fig. 1B, C). In thermal and cold hyperalgesia, SNL surgery resulted in a significant reduction in paw withdrawal latency (Fig. 1D, E). HE staining of the spinal cord of the mice showed the presence of significant inflammatory cell infiltration (Fig. 1F). We then extracted DRG neurons from modeled mice and obtained DRG-Exo by differential ultracentrifugation. Under TEM, DRG-Exo were cup-shaped (Fig. 1G), and NTA showed that the average size of DRG-Exo was 101.49 ± 10.06 nm (Fig. 1H). Western blot confirmed the expression of surface markers CD9, CD63, and CD81 in extracted DRG-Exo with the absence of Calnexin and GM130 (Fig. 1I).

Upon the treatment of DRG-Exo, the mice showed a significantly increased frequency of paw withdrawal in response to both 0.07 g and 0.4 g von Frey filament stimulation (Fig. 1J, K). DRG-Exo-treated mice also exhibited an increased response to thermal and cold hyperalgesia with a further reduction in paw withdrawal latency (Fig. 1L, M). HE staining also showed a further increase in inflammatory cell infiltration in the spinal cord of mice (Fig. 1N), suggesting that DRG-Exo caused an exacerbation of NP.

### DRG-Exo promote neuroinflammation through the activation of microglia

**Fig. 2:**
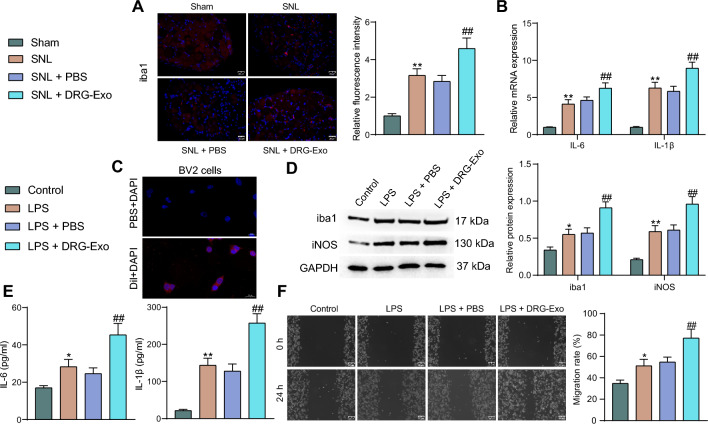
DRG-Exo promote neuroinflammation in SNL-induced mice by activating microglial activation. **a** Expression of iba1, a marker of microglial activation in the spinal cord of mice by immunofluorescence. **b** Expression of IL-6 and IL-1β in the spinal cord of mice by RT-qPCR. **c** The uptake of DRG-Exo by BV2 cells. **d** Activation marker iba1 and pro-inflammatory marker iNOS expression in LPS and DRG-Exo-treated BV2 cells. **e** The levels of IL-6 and IL-1β in LPS and DRG-Exo-treated BV2 cells using ELISA. **f** The migration capacity of BV2 cells after LPS and DRG-Exo treatment was measured using cell scratch assay. Values are expressed as mean ± SD. *p < 0.05, **p < 0.01 vs the control group and ##*p* < 0.01 vs the SNL + PBS or LPS + PBS group by one-way (**a**, **e**, **f**) or two-way ANOVA (**b**, **d**). The cell experiment was independently repeated three times

We performed immunofluorescence staining for iba1, a marker of microglia activation [14, 17], in the spinal cord of SNL mice. It was found that iba1 was significantly increased in the spinal cord of SNL mice, which was further promoted by DRG-Exo treatment (Fig. 2A). IL-6 and IL-1β level evaluation in the spinal cord of mice using RT-qPCR confirmed the onset of neuroinflammation caused by SNL, as evidenced by a significant increase in mRNA levels of IL-6 and IL-1β. DRG-Exo further enhanced the inflammatory response with an augment in IL-6 and IL-1β expression in the presence of SNL (Fig. 2B).

Next, we investigated the effect of DRG-Exo on microglial activation in BV2 cells. BV2 cells were pre-treated with LPS, followed by treatment of DRG-Exo (with PBS as control). Exo labeling assays confirmed the uptake of DRG-Exo by BV2 cells (Fig. 2C). Western blot assays on activation marker iba1 and pro-inflammatory marker iNOS in BV2 cells showed that LPS expedited the expression of iba1 and iNOS in BV2 cells, and DRG-Exo contributed to a further increase in iba1 and iNOS expression (Fig. 2D). ELISA assay of IL-6 and IL-1β in the cell supernatant showed that LPS stimulated IL-6 and IL-1β levels secreted by BV2 cells, which was further promoted by DRG-Exo (Fig. 2E). Cell scratch assays demonstrated that LPS encouraged the BV2 cell migration and the DRG-Exo administration prompted the migratory capacity of BV2 cells (Fig. 2F).

### DRG-Exo promote microglial activation through the delivery of miR-16-5p

**Fig. 3:**
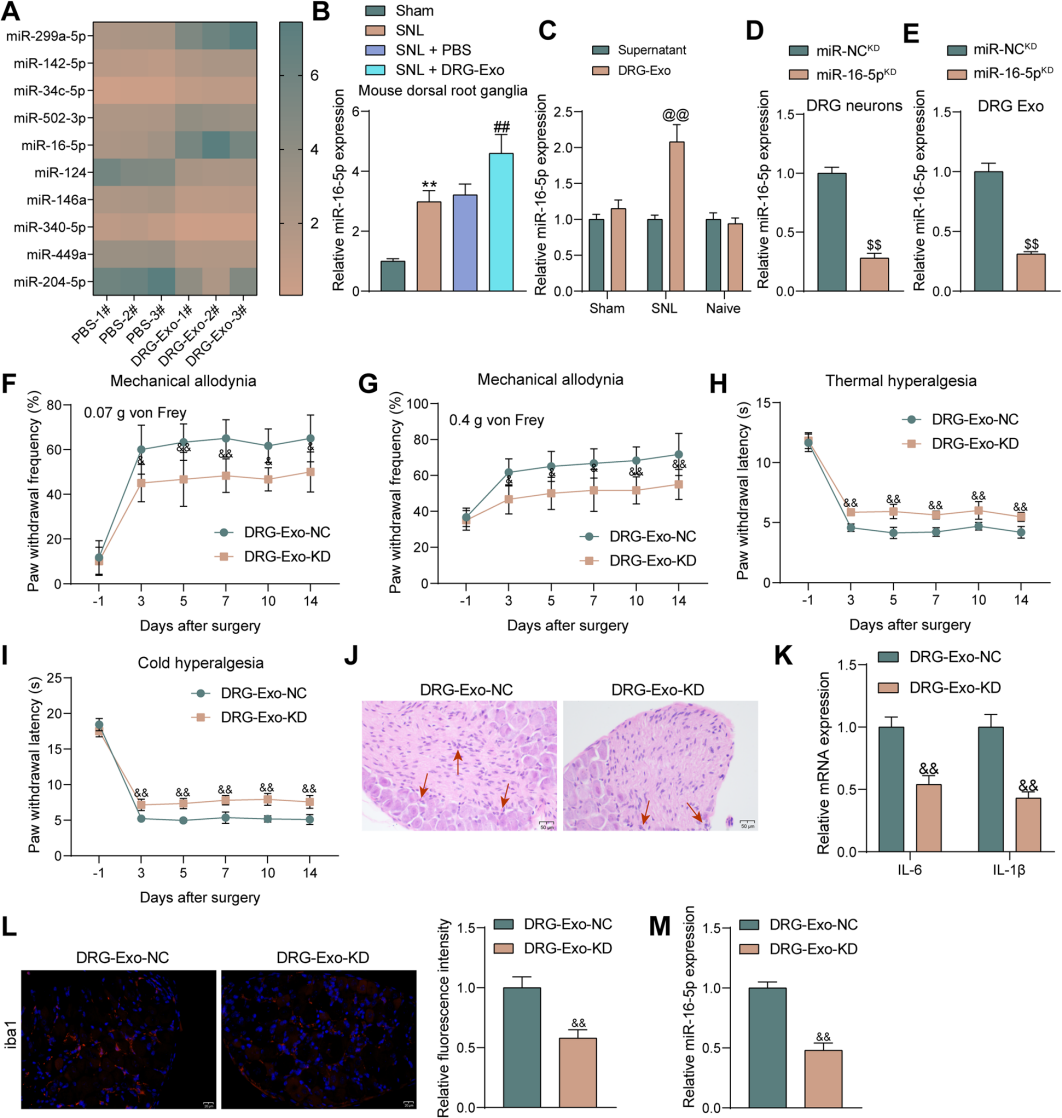
DRG-Exo are involved in the development of NP through the delivery of miR-16-5p. **a** Microarray analysis of differentially enriched miRNAs in BV2 cells after DRG-Exo treatment. **b** miR-16-5p expression in the spinal cord of SNL and DRG-Exo-treated mice using RT-qPCR. **c** RT-qPCR detection of miR-16-5p enrichment in the sham-operated, SNL-treated, and naive mice-derived DRG-Exo. **d** miR-16-5p expression in DRG neurons upon miR-16-5p^KD^ or miR-NC^KD^ using RT-qPCR. **e** miR-16-5p expression in DRG-Exo-KD or DRG-Exo-NC using RT-qPCR. Assessment of mechanical allodynia response in mice treated with DRG-Exo-KD or DRG-Exo-NC using 0.07 g (**f**) or 0.4 g (**g**) von Frey filaments at different time points before and after SNL surgery, respectively. **h** The responses of mice treated with DRG-Exo-KD or DRG-Exo-NC to thermal hyperalgesia were evaluated at different time points before and after SNL surgery. **i** The responses of mice treated with DRG-Exo-KD or DRG-Exo-NC to cold hyperalgesia were assessed at different time points before and after SNL surgery, respectively. **j** Inflammatory infiltration in the spinal cord (L4) of the mice treated with DRG-Exo-KD or DRG-Exo-NC was observed using HE staining. **k** The mRNA expression of IL-6 and IL-1β in the spinal cord by RT-qPCR. **l** Expression of iba1, a marker of microglia activation in the spinal cord by immunofluorescence. **m** Expression of miR-16-5p in the spinal cord by RT-qPCR. Values are expressed as mean ± SD. **p < 0.01 vs the sham group; ##*p* < 0.01 vs the SNL + PBS group; @@*p* < 0.01 vs the supernatant group; $$*p* < 0.01 vs the miR-NC.^KD^ group; &&*p* < 0.01 *vs* the DRG-Exo-NC group by unpaired *t-test* (**d**, **e**, **l**, **m**), one-way (**b**) or two-way ANOVA (**c**, **f**, **g**, **h**, **i**, **k**)

We firstly performed miRNA-based microarray analysis on PBS- or DRG-Exo-treated BV2 cells (Fig. 3A). Among the ten differentially expressed miRNAs induced by DRG-Exo, miR-16-5p has been reported to be as a key regulator in the progression of NP [16]. However, the mechanism of miR-16-5p in NP has not been clarified yet. The expression of miR-16-5p was significantly increased in the spinal cord of SNL-treated mice, and DRG-Exo treatment further increased the expression of miR-16-5p (Fig. 3B). We further extracted DRG-Exo from sham-operated mice and naive mice, and RT-qPCR was conducted to detect the enrichment of miR-16-5p in these three kinds of DRG-Exo. It was revealed that miR-16-5p was significantly enriched in the DRG-Exo of SNL-treated mice. Moreover, its enrichment was not found in DRG-Exo of sham-operated and naïve mice (Fig. 3C), suggesting that DRG-Exo may contribute to NP through the delivery of miR-16-5p. Therefore, we treated DRG with miR-16-5p inhibitor or NC inhibitor (named miR-16-5p^KD^ or miR-NC^KD^ thereafter). RT-qPCR for miR-16-5p expression in DRG and DRG-Exo showed a significant decrease in miR-16-5p expression (Fig. 3D, E). The Exo extracted from treated DRG were named DRG-Exo-NC or DRG-Exo-KD.

We then treated SNL mice with DRG-Exo-NC or DRG-Exo-KD and found a significant decrease in paw withdrawal frequency in response to mechanical allodynia (Fig. 3F, G), and an increase in paw withdrawal latency in response to thermal and cold hyperalgesia (Fig. 3H, I). Inflammatory infiltration was improved in the spinal cord of mice treated with DRG-Exo-KD (Fig. 3J). Furthermore, mRNA expression of IL-6 and IL-1β in the spinal cord was reduced (Fig. 3K), and microglial activation was significantly hampered (Fig. 3L). Detection of miR-16-5p expression in the spinal cord of mice showed a significant decrease in miR-16-5p expression after the delivery of DRG-Exo-KD (Fig. 3M).

### HECTD1 is a putative target of miR-16-5p in NP

**Fig. 4:**
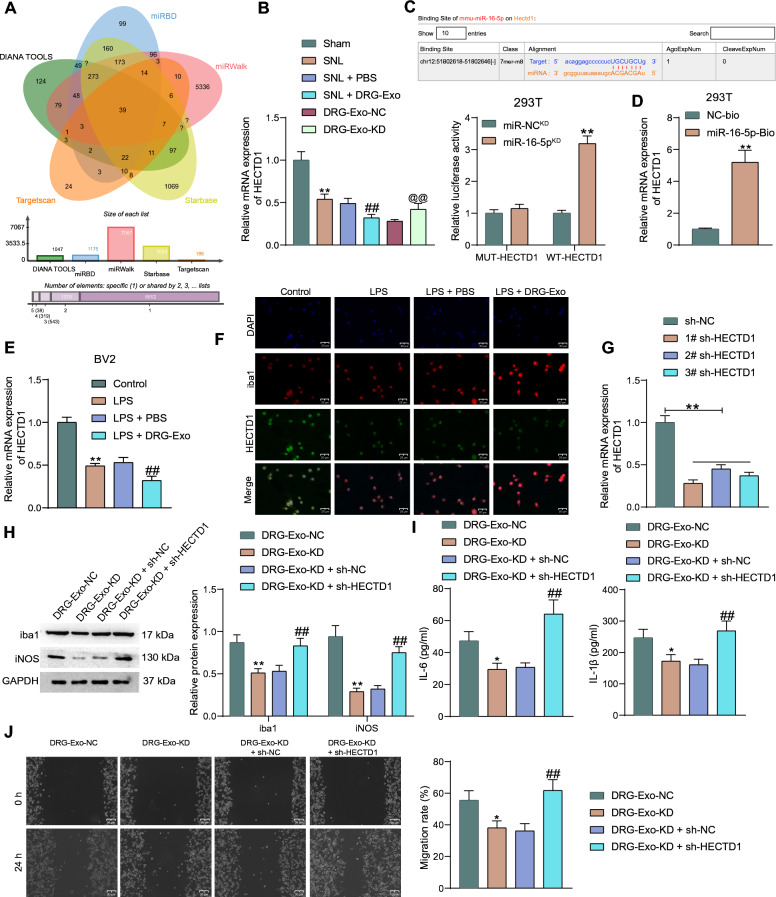
miR-16-5p interacts with HECTD1 to promote microglial activation. **a** The intersection of predicted downstream targets of miR-16-5p in Starbase, Targetscan, miRWalk, miRBD, and DIANA TOOLS databases. **b** Detection of HECTD1 expression in the spinal cord of mice by RT-qPCR. **c** Binding sites of miR-16-5p and HECTD1 predicted by the Starbase database and the luciferase activity in 293T cells with WT and MUT HECTD1 binding sites using dual-luciferase assays. **d** The expression of HECTD1 mRNA in biotin-coupled miR-16-5p pull-down complexes using an RNA pull-down assay. **e** Detection of HECTD1 mRNA expression in LPS- and DRG-Exo-treated BV2 cells. **f** Co-localization of HECTD1 and iba1 in LPS and DRG-Exo-treated BV2 cells using immunofluorescence staining. **g** Detection of HECTD1 mRNA expression after knockdown of HECTD1 in BV2 cells using RT-qPCR. **h** Iba1 and iNOS protein expression in BV2 cells after treatment with DRG-Exo-KD and HECTD1 knockdown. **i** IL-6 and IL-1β levels in BV2 cells after treatment with DRG-Exo-KD and HECTD1 knockdown using ELISA. **j** Cell migration ability in BV2 cells after treatment with DRG-Exo-KD and HECTD1 knockdown detected by cell scratch assay. Values are expressed as mean ± SD and analyzed using unpaired t-test or one-way/two-way ANOVA. *p < 0.05, **p < 0.01 vs the sham, miR-NC^KD^, NC-Bio, control, sh-NC, or DRG-Exo-NC group; ##*p* < 0.01 vs the SNL + PBS, LPS + PBS, or DRG-Exo + sh-NC group; @@*p* < 0.01 vs the DRG-Exo-NC group by unpaired *t-test* (**d**), one-way (**b**, **e**, **g**, **i**, **j**) or two-way ANOVA (**c**, **h**). Cell experiments were independently repeated three times

To search for the downstream targets of miR-16-5p in NP, we queried Starbase (https://starbase.sysu.edu.cn/index.php), Targetscan (https://www.targetscan.org/vert_80/), miRWalk (http://mirwalk.umm.uni-heidelberg.de/), miRBD (http://mirdb.org/mirdb/index.html), DIANA TOOLS (https://diana.e-ce.uth.gr/) databases. A total of 39 intersecting target genes were identified (Fig. 4A). Among the intersecting targets, we noted HECTD1 since it is closely associated with neuroinflammation [18].

We found that HECTD1 expression was significantly decreased in the spinal cord of SNL-treated mice relative to the control mice. Furthermore, HECTD1 expression was further downregulated by DRG-Exo, and restored after miR-16-5p^KD^ in DRG-Exo (Fig. 4B). We predicted the binding site of miR-16-5p to HECTD1 in the Starbase database and constructed MUT and WT sequences for the binding site. The results of dual-luciferase assays showed that miR-16-5p^KD^ enhanced the luciferase activity of 293T cells with the WT binding site, but had an insignificant effect on the MUT (Fig. 4C). In RNA pull-down assays, biotin-labeled complexes were enriched with HECTD1 mRNA (Fig. 4D).

In terms of microglial activation, HECTD1 expression was significantly reduced in LPS-treated BV2 cells, and HECTD1 expression was further suppressed after DRG-Exo treatment (Fig. 4E). Immunofluorescence analysis showed that HECTD1 co-localized with iba1 in BV2 cells, and the expression of HECTD1 gradually decreased with the activation of iba1 (Fig. 4F). We knocked down HECTD1 in BV2 cells using shRNAs targeting HECTD1. After RT-qPCR to verify the knockdown efficiency (Fig. 4G), BV2 cells challenged with LPS were further subjected to DRG-Exo-KD alone or in combination with sh-HECTD1 (sh-NC as a control) treatments. DRG-Exo-KD treatment resulted in a significant reduction of iba1 and iNOS expression in BV2 cells, while iba1 and iNOS expression was restored after sh-HECTD1 in BV2 cells (Fig. 4H). The levels of IL-6 and IL-1β in the cell supernatant were also significantly decreased by DRG-Exo-KD and partially upregulated after the knockdown of HECTD1 in BV2 cells (Fig. 4I). BV2 cell migration, consistently was impaired under the influence of DRG-Exo-KD, which was reversed by knockdown of HECTD1 (Fig. 4J).

### Ubiquitination modification of HSP90 by HECTD1 inhibits the expression of HSP90

**Fig. 5:**
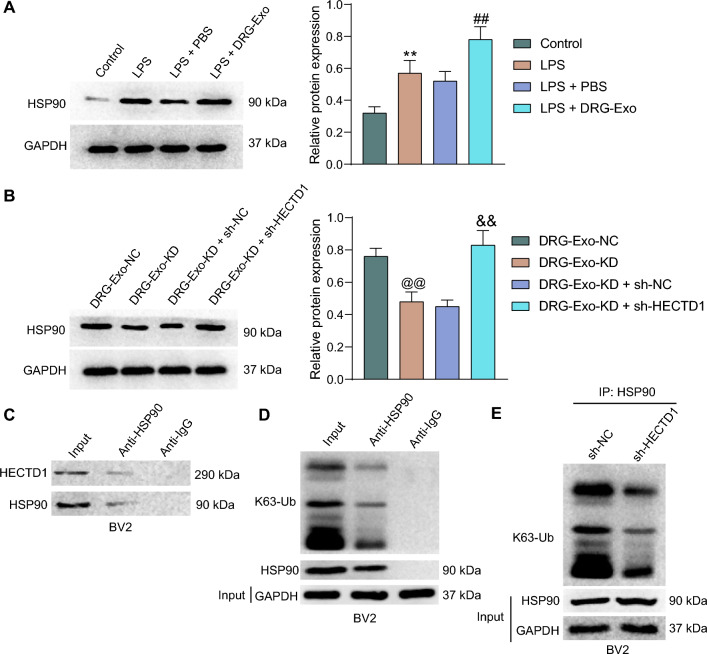
Ubiquitination modification of HSP90 by HECTD1 inhibits the expression of HSP90. **a** HSP90 protein expression in BV2 cells treated with LPS and DRG-Exo using western blot. **b** HSP90 protein expression in BV2 cells after treatment with DRG-Exo-KD and HECTD1 knockdown using western blot. The binding relationship between HSP90 and HECTD1 (**c**) or K63-Ub (**d**) in BV2 cells using Co-IP assays. **e** Ubiquitination levels after knockdown of HECTD1 in BV2 cells using ubiquitination immunoprecipitation assays. Values are expressed as mean ± SD. **p < 0.01 vs the control group; ##*p* < 0.01 vs the LPS + PBS group; @@*p* < 0.01 vs the DRG-Exo-NC group; &&*p* < 0.01 vs the DRG-Exo-KD + sh-NC group by one-way ANOVA. Cell experiments were independently repeated three times

In BV2 cells, we found that LPS treatment promoted the protein expression of HSP90, and DRG-Exo enhanced this effect (Fig. 5A). In contrast, the protein expression of HSP90 was decreased when DRG-Exo-KD was applied, and the knockdown of HECTD1 in BV2 cells led to the increased protein expression of HSP90 in the presence of DRG-Exo-KD (Fig. 5B). Co-IP assays confirmed the binding between HECTD1 and HSP90 in BV2 cells. Similarly, there was a binding relation between HSP90 and K63-ub (Fig. 5C, D). In the ubiquitinated immunoprecipitation assay of BV2 cells, knockdown of HECTD1 inhibited the interaction between HSP90 and K63-Ub (Fig. 5E), suggesting that HECTD inhibited HSP90 expression through ubiquitination of HSP90 on the K63 site.

### Overexpression of HSP90 or silencing of HECTD1 promotes microglial activation in NP

**Fig. 6:**
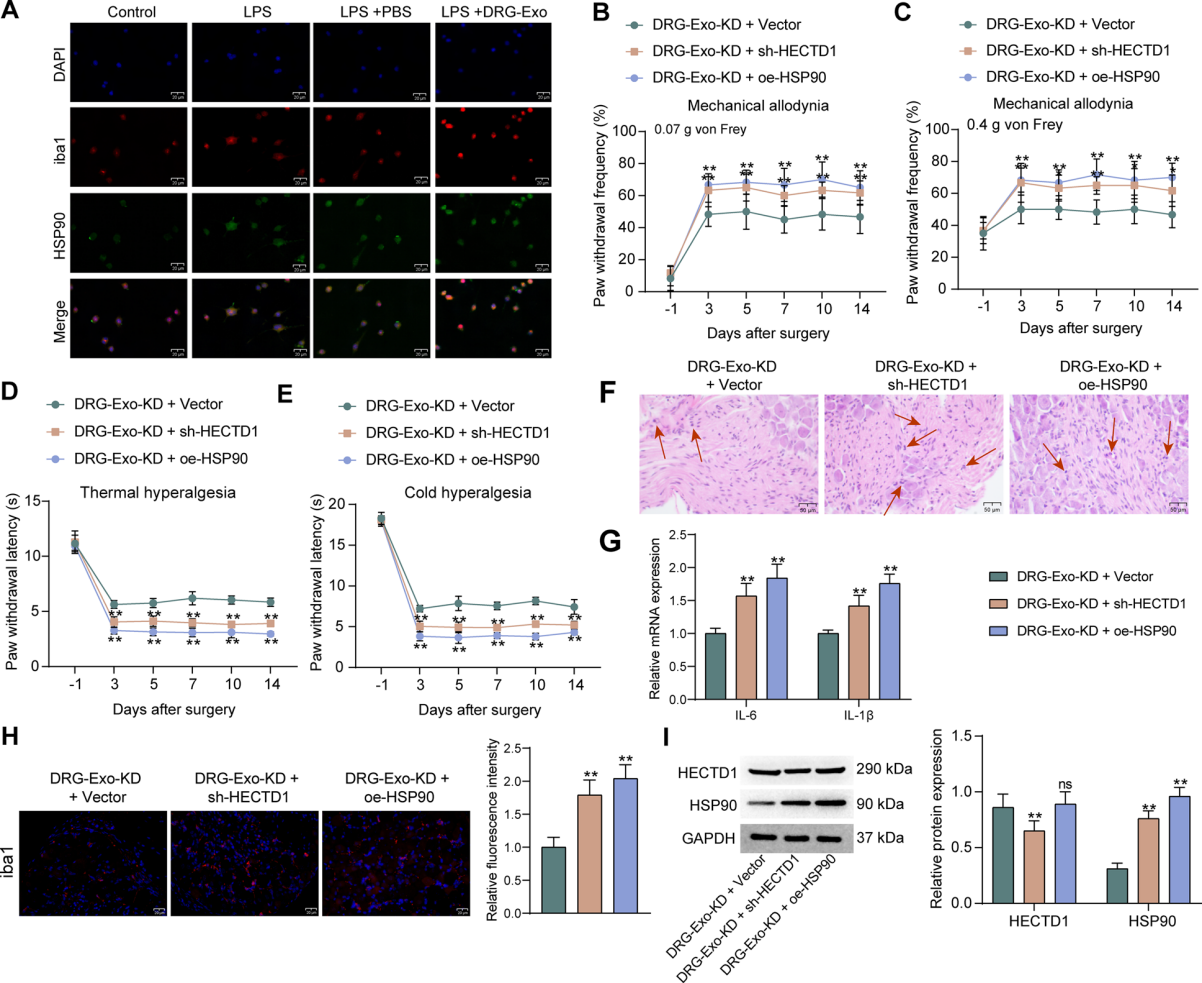
Depletion of HECTD1 or overexpression of HSP90 promotes microglial activation in NP. **a** Co-localization of iba1 and HSP90 in LPS and DRG-Exo-treated BV2 cells detected by dual immunofluorescence. Assessment of mechanical allodynia response in mice treated with DRG-Exo-KD + sh-HECTD1/oe-HSP90 using 0.07 g (**b**) or 0.4 g (**c**) von Frey filaments at different time points before and after SNL surgery, respectively. **d** The responses of mice treated with DRG-Exo-KD + sh-HECTD1/oe-HSP90 to thermal hyperalgesia were evaluated at different time points before and after SNL surgery. **e** The responses of mice treated with DRG-Exo-KD + sh-HECTD1/oe-HSP90 to cold hyperalgesia were assessed at different time points before and after SNL surgery, respectively. **f** Inflammatory infiltration in the spinal cord (L4) of the mice treated with DRG-Exo-KD + sh-HECTD1/oe-HSP90 was observed using HE staining. **g** Expression of IL-6 and IL-1β in the spinal cord of mice by RT-qPCR. **h** Expression of iba1, a marker of microglia activation in the spinal cord by immunofluorescence. **i** The protein expression of HECTD1 and HSP90 in the spinal cord by western blot. Values are expressed as mean ± SD and analyzed using one-way/two-way ANOVA. **p < 0.01 vs the DRG-Exo-KD + Vector group by one-way (**h**) or two-way ANOVA (**b**, **c**, **d**, **e**, **g**, **i**); *ns* indicates not significant

To verify the effect of HSP90 on the microglial activation-mediated NP, co-localization analysis of iba1 and HSP90 was first performed in BV2 cells using dual-immunofluorescence analysis. An increase in the fluorescence intensity of HSP90 was observed with microglial activation, which was more pronounced upon DRG-Exo treatment (Fig. 6A). Then, AAV harboring oe-HSP90 or sh-HECTD1 was injected at the spinal cord in mice in the presence of DRG-Exo-KD. Interestingly, we found that knockdown of HECTD1 or overexpression of HSP90 at the spinal cord reversed the alleviating effect of DRG-Exo-KD, as evidenced by increased frequency of paw withdrawal in response to mechanical allodynia (Fig. 6B, C) and shortened paw withdrawal latency in response to thermal and cold hyperalgesia (Fig. 6D, E). Increased inflammatory infiltration in the spinal cord of mice (Fig. 6F) and significantly increased expression of IL-6 and IL-1β were found following sh-HECTD1 or oe-HSP90 treatment (Fig. 6G). Meanwhile, significantly increased activation of microglia was observed using iba1-immunofluorescence upon AAV injection (Fig. 6H). In addition, the knockdown of HECTD1 increased the protein expression of HSP90, whereas overexpression of HSP90 did not affect the expression of HECTD1 (Fig. 6I).

## Discussion

All neural cells, including neurons, astrocytes, oligodendrocytes, and microglia, release extracellular vesicles comprising Exo and ectosomes both in normal and pathological conditions [19]. For instance, miR-124-3p transmitted by neuron-derived Exo can protect traumatically injured spinal cord by suppressing the activation of neurotoxic microglia [20]. In the present study, we observed that DRG neuron-derived Exo deteriorated SNL-induced mechanical allodynia, thermal and cold hyperalgesia, and neuroinflammation possibly through the delivery of miR-16-5p. Furthermore, we demonstrated that miR-16-5p inhibitor transmitted by DRG-Exo exerted an antinociceptive effect, at least partly by suppressing the HECTD1-mediated HSP90 ubiquitination in DRG.

Traditionally, glia, composed of astrocytes, oligodendrocytes, ependymocytes, and microglia, were disregarded as the structural support across the brain and spinal cord, in striking contrast to neurons, always considered critical players in CNS functioning [21]. DRG is one of the most critical structures in sensory signaling and regulation, along with pain transmission [22]. Simeoli et al*.* reported that DRG neurons-derived Exo released by capsaicin were phagocytosed by macrophages in which an increase in miR-21-5p expression promoted a pro-inflammatory phenotype [23], indicating the possible communication between DRG-Exo with recipient cells. However, the crosstalk between DRG-Exo and microglial activation has been rarely investigated. We found here that the administration of DRG-Exo enhanced the iba1 expression in the DRG of SNL-induced mice and the expression of iba1 and iNOS in BV2 cells treated with LPS. Iba1 represents a cytoskeleton protein specific for microglia and macrophages [24]. Given that cells stained positive for Iba-1 could potentially be satellite glial cells or infiltrating macrophages, our conclusion needs to be further confirmed in future studies. iNOS is inducible and predominantly expressed by immune cells, including microglia where it plays indispensable roles in inflammation and the defense against pathogens [25]. Although a non-specific uptake is shared by all cell types, specific targeting to recipient cells is paramount to exert the function of Exo which is mediated by the surface composition of the Exo and the conservation of tropism between donor and recipient cells. This cellular signature conserved in the secreted Exo acts as a recognition motif for uptake by the same recipient cell types in vitro and in vivo [26]. Therefore, the successful uptake of DRG-Exo by BV2 cells in vitro verifies our in vivo observation of iba1 upregulation. Still, whether DRG-Exo can be up-taken by microglia through the same mechanism awaits further studies. Microglial cell migration has been related to proinflammatory responses [27, 28]. In the present study, we also verified the pro-migratory effects of DRG-Exo.

MiR-16 expression was significantly upregulated in the spinal cord of rats with chronic constriction injury, and intrathecal injection of miR-16 inhibitor significantly attenuated the mechanical allodynia and thermal hyperalgesia and downregulated the expression of IL-1β in the spinal cord of rats [29]. Macrophage-derived Exo delivering miR-16-5p aggravated atherosclerosis progression in mice via facilitating inflammatory response and oxidative stress [30]. Here, we also found that the upregulation of miR-16-5p in DRG was responsible for the pro-inflammatory effects of DRG-Exo in NP since miR-16-5p knockdown in DRG using miR-16-5p inhibitor successfully inhibited the effects of DRG-Exo in vivo and in vitro.

To probe the downstream targets of miR-16-5p in NP, we integrated several bioinformatics websites for prediction. A total of 39 targets were obtained, and HECTD1 drew our attention since it has been associated with LPS-induced astrocyte activation, thereby serving as a potential therapeutic strategy for neuroinflammation induced by LPS [18]. Here, we verified the binding relation between miR-16-5p and HECTD1. Likewise, miR-186-5p knockdown mitigated cell damage by increasing HECTD1 levels in oxygen–glucose deprivation-treated human brain microvascular endothelial cells [31]. However, the functional role of HECTD1 in NP has not been well described. Intriguingly, HECTD1 has been identified as a target of miR-9 during the microglial activation, which was elicited through the HSP90 ubiquitination [32]. Epigenetic modifications, including histone modifications (involving methylation, acetylation, and ubiquitination), DNA methylation, and noncoding RNA regulation have been recently implicated in the development of NP [33]. The ubiquitination of proteins comprises several steps: firstly, the E1 ubiquitin-activating enzyme induces ubiquitin, and then the E2 ubiquitin-conjugating enzyme∼Ub interacts with E3 ubiquitin ligase; finally, the E3 ligase transfers the E2-bound ubiquitin to a substrate and mediates the ubiquitination [34]. HECTD1 is such an E3 ubiquitin ligase and HSP90, a chaperone protein capable of enhancing the migration of multiple cell types, is one of its substrates [35]. Here, we validated the HSP90 modification by HECTD1 at the K63. HSP90 inhibition has emerged as a possible target in treating an array of diseases, especially neurodegenerative diseases [36]. The data obtained from our rescue experiments also showed that overexpression of HSP90 and silencing of HECTD1 overturned the effects of DRG-Exo with miR-16-5p inhibitor on mechanical allodynia and thermal and cold hyperalgesia and the neuroinflammation.

## Conclusion

**Fig. 7:**
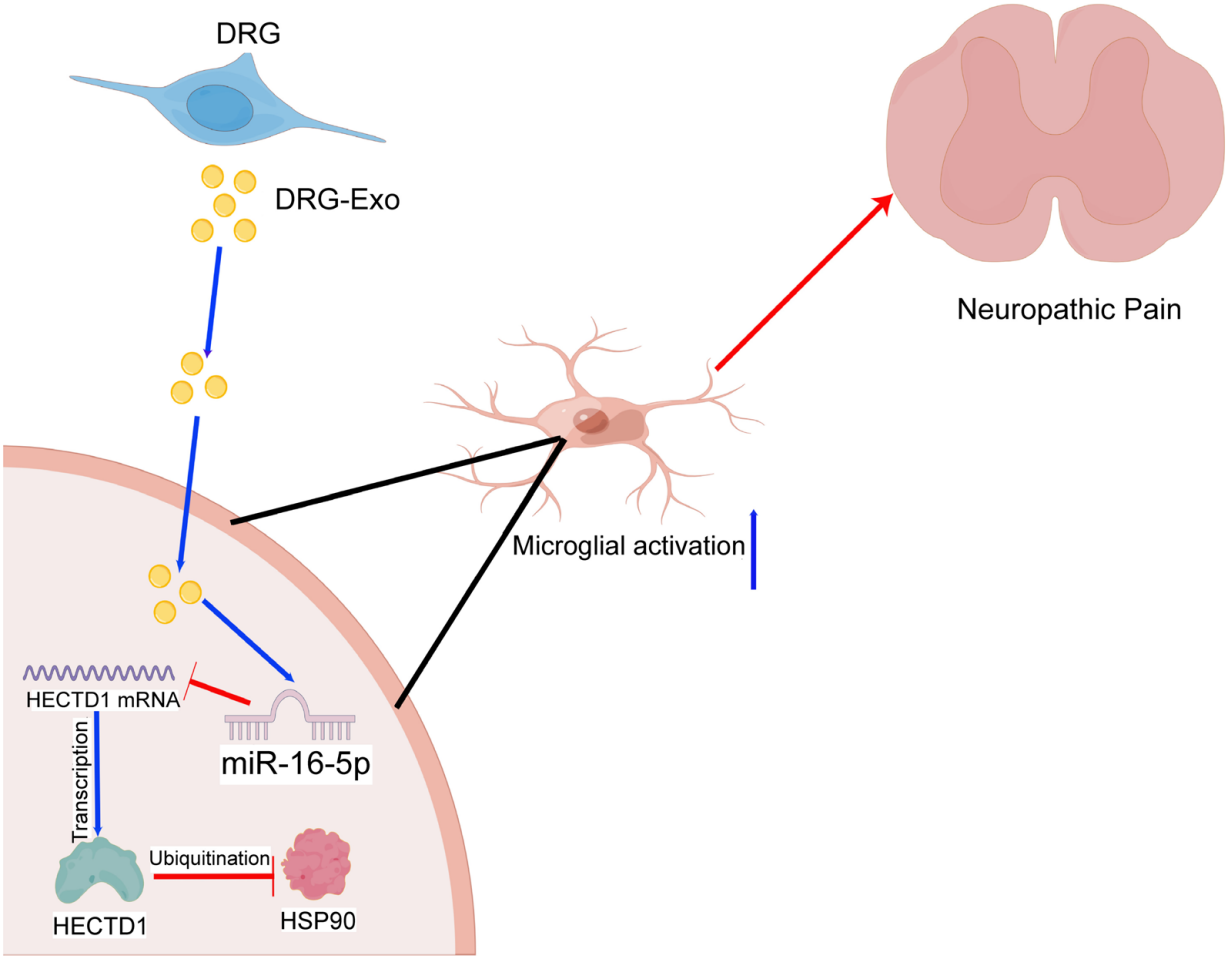
The scheme illustrates the mechanism of DRG-Exo in NP (by Figdraw, https://www.figdraw.com/static/index.html). Exosomal delivery of miR-16-5p by DRG neurons interacts with HECTD1 to regulate ubiquitination levels of HSP90, thereby promoting microglial activation in NP

In summary, our findings demonstrated that intrathecally injected DRG-Exo exacerbated the SNL-induced NP in mice via miR-16-5p delivery. The present research confirmed that miR-16-5p released from DRG-Exo encouraged the microglial activation and promoted the production of the pro-inflammatory cytokines IL-1β and IL-6 via HECTD1-mediated HSP90 ubiquitination (Fig. 7). Our findings provide a novel mechanism through which microglial activation induces NP.

## Materials and methods

### Animals

All animal procedures were conducted according to protocols approved by the Institutional Animal Care and Use Committee of Shengjing Hospital of China Medical University and consistent with the US National Institutes of Health guidelines. All efforts were made to minimize animal suffering and reduce the number of mice used. Adult (6–8 weeks) and naive (3–5 weeks) male C57BL/6J mice (Beijing Vital River Laboratory Animal Technology Co., Ltd., Beijing, China) were housed in clean, pathogen-free cages (no more than 5 mice per cage). A 12–12 h lights on–off cycle was maintained in an environmentally controlled room (23 ± 1 ℃, 55% humidity). Food and water were provided ad libitum.

We performed the L4 SNL modeling in mice according to the instructions reported [37]. The mice were anesthetized with isoflurane and fixed in the prone position. A skin incision was made on the dorsolateral aspect of the lower back using a sterilized scalpel to remove the L5 transverse process and expose the unilateral L4 spinal nerve. After the isolation of the L4 spinal nerve, the nerve was ligated using a 7–0 silk suture, followed by transection of the ligated nerve at the distal end of the ligature under an operating microscope. Finally, the mouse wound was sutured. The mice in the sham group underwent the same operation as the SNL-operated mice except for nerve ligation. Behavioral tests were performed on mice on the first preoperative day, and the 3rd, 5th, 7th, 10th, and 14th postoperative days, respectively. The mice were euthanized by intraperitoneal injection of 150 mg/kg sodium pentobarbital after the 14th day.

### Isolation, culture, and treatment of DRG neurons

After the euthanasia of (sham-operated mice, mice 14 d after SNL, and naïve mice) by intraperitoneal injection of 150 mg/kg sodium pentobarbital, the spinal cord was dissected. L4 DRG were isolated and collected in Dulbecco's modified Eagle's medium (DMEM)/F12 medium containing 90% DMEM, 10% FBS, 100 U/mL penicillin, and 100 mg/mL streptomycin (Thermo Fisher Scientific Inc., Waltham, MA, USA). After the removal of the connective tissue, DRG were enzymatically dissociated in trypsin and collagenase D at room temperature for 30 min. Calf serum was then added to neutralize collagenase, the DRG were ground in a glass pipette. The cell suspension was filtered through a 70 μm nylon mesh filter to remove undissociated cells with myelin. The cells were centrifuged at 300 *g* for 10 min and resuspended in a fresh medium. The cells were resuspended in 1 mL of F-12 and then spun through a column with 15% essentially fatty acid-free bovine serum albumin (A9205; Sigma-Aldrich, St. Louis, MO, USA) in F-12 at 900 g for 10 min. DRG (0.2 mL/per well) were plated onto poly-L-Lysine pre-coated 12 mm glass-bottom tissue culture dish or 12 mm coverslips in 24 well culture plates at 37 °C and 5% CO_2_.

Primary DRG neurons extracted from SNL mice were collected, and the endogenous maturation of miR-16-5p in DRG neurons was inhibited using a miR-16-5p inhibitor (designed and constructed by Shanghai GenePharma Co., Ltd., Shanghai, China). Briefly, primary DRG neurons were seeded in 24-well plates. miR-16-5p inhibitor (named miR-16-5p^KD^) or control (named miR-NC^KD^) was transfected into cultured DRG neurons using lipofectamine 2000. After transfection for 6 h, the medium was replaced with fresh medium. After 24 h of incubation, the expression of miR-16-5p in DRG neurons was detected by RT-qPCR to verify the transfection efficiency.

### The extraction and in vivo injection of DRG-Exo

Primary DRG neurons extracted from sham-operated mice, SNL-treated mice, and naïve mice were cultured in an Exo-free medium, and the cell culture supernatant was collected and stored at −80 °C. The cell culture supernatants were centrifuged at 300 g for 10 min, 2000 g for 10 min, and 10,000 g for 0.5 h at 4 °C. The obtained supernatant was subjected to two rounds of ultracentrifugation at 100,000 g for 1 h at 4 °C using an ultracentrifuge to harvest the precipitate for resuspension in phosphate-buffered saline (PBS). Total exosomal protein was measured by the bicinchoninic acid (BCA) protein concentration method, and exosomal surface marker proteins CD9 (1:1000, ab307085, Abcam, Cambridge, UK), CD63 (1:1000, ab216130, Abcam), CD81 (1:1000, ab232390, Abcam), and the contaminants calnexin (1:5000, PA5-34754, Thermo Fisher) and GM130 (1:1000, PA5-95727, Thermo Fisher) in 200 μL solution containing Exo were detected using western blot. Transmission electron microscopy (TEM) was used to observe the morphology of DRG-Exo.

Nanoparticle Tracking Analysis (NTA) was used to measure the diameter of DRG-Exo. The particle size and concentration of DRG-Exo were analyzed with the Malvern nanoparticle tracking analyzer NanoSightNS300. The isolated DRG-Exo precipitates were resuspended with PBS and slowly injected into the cuvette, which was placed into the instrument and instrumented according to standard operating procedures.

For DRG-Exo injection, SNL-operated mice received intrathecal injections of 5 μL DRG-Exo extracted from SNL-treated mice (1 μg/μL) daily for 3 days starting on the first day after SNL, and control mice received equal doses of PBS (the SNL + PBS group).

### Adeno-associated virus (AAV) injection

To investigate the effect of HECTD1 and HSP90 expression alteration, we injected AAV vectors containing sh-HECTD1 and overexpressed HSP90 plasmids as well as control empty vectors (titer ≥ 10^13^) into exposed the L4 segments of the spinal cord by microinjection 5 d before SNL, followed by SNL modeling and DRG-Exo treatment.

### Behavioral tests

Mice were tested behaviorally on the first preoperative day and the 3rd, 5th, 7th, 10th, and 14th postoperative days as described previously [38]. For the mechanical allodynia, the mice were placed in the cage net platform and stimulated with two calibrated von Frey filaments (0.07 g and 0.4 g) for 1 s on the hind paws. The stimulation was repeated 10 times, with an interval of 5 min before and after each stimulation, during which each paw withdrawal was recorded. The percentage of the number of paw withdrawals in the 10 trials was taken as the frequency of paw withdrawal (%).

For the thermal hyperalgesia, the mice were placed in a Plexiglas box with a glass plate at the bottom, and a Model 336 Analgesic Meter (IITC Life Science Inc., Woodland Hills, CA, USA) was used to irradiate the middle of the plantar surface of the hind paw from the hole of the light box through the glass plate. The mice lifted their hind paws immediately when they were thermally stimulated by light irradiation. The duration from the start of light irradiation to the time the mice lifted their hind paws was recorded and defined as the paw withdrawal latency (s). Five trials with 5-min intervals were carried out, and the maximum beam time was 20 s to avoid damage to the mice.

For the cold hyperalgesia, the mice were placed on a cold aluminum plate maintained at a temperature of 0 °C. The duration of the hind paw withdrawal or paw licking in mice recorded from the beginning of the experiment was defined as the paw withdrawal latencies to cold (s). The experiment was repeated three times with 10 min intervals between each time, and the same maximum stimulation time of 20 s was set to avoid damage to the mice.

### Hematoxylin–eosin (HE) staining

HE staining was used to examine the inflammatory infiltration of mouse spinal cord tissues. After deep anesthesia with isoflurane, the hearts of mice were perfused with 0.1 M PBS and 4% paraformaldehyde. The mouse spinal cord tissues (L4) were dissected at 4 °C, fixed in 4% paraformaldehyde for 8 h, dehydrated using 30% sucrose solution, and made into 20-μm sections after paraffin-embedding. The sections were dewaxed and rehydrated with xylene and gradient alcohol, followed by a 2-min hematoxylin staining. Afterward, the sections were treated using hydrochloric acid for 2 s and washed. Then, the eosin staining solution was added for a 2-min incubation. Subsequently, the sections were dried and photographed to analyze the inflammatory infiltration.

### Immunofluorescence staining

Paraffin-embedded spinal cord sections were dewaxed and rehydrated, sealed, and permeabilized in 5% goat serum and 0.3% Triton X-100 in PBS, respectively. After washing, the sections were incubated with iba1 antibody (1:800, 17198, Cell Signaling Technologies, Beverly, MA, USA) at 4 °C overnight and with goat anti-rabbit IgG H&L (Alexa Fluor^®^ 647) (1:500, ab150083, Abcam) at room temperature for 2 h, blocked, and observed for fluorescence using a laser confocal microscope.

### Cell culture and treatment

Mouse microglia BV2 (CL-0493A) and 293T cells (CL-0005) were purchased from Procell (Wuhan, Hubei, China) and cultured in DMEM (Thermo Fisher) containing 10% FBS and 100 U/mL penicillin + 100 mg/mL streptomycin at 37 ℃ with 5% CO_2_ after activation. To assess the effect of DRG-Exo on microglia activation, BV2 cells were first stimulated with 100 ng/mL LPS for 2 h, followed by the addition of 100 μg/mL DRG-Exo. The cells were collected after 24 h of incubation for subsequent experiments.

The cultured BV2 cells were seeded into 6-well plates, and 10 μL of AAV vector with sh-HECTD1 was added to each well for cell infection. After 6 h of culture, the culture medium was replaced with a fresh medium, and the HECTD1 expression was verified by RT-qPCR after 48 h of culture.

### The uptake of DRG-Exo assay

DRG-Exo were labeled with Dil solution (D3911, Thermo Fisher) according to the manufacturer's instructions or treated with the same volume of PBS as control. After that, the BV2 cells were co-cultured with 100 μg/mL DRG-Exo for 24 h and fixed in 4% paraformaldehyde. The BV2 cell nuclei were labeled with DAPI, and the uptake of Dil-labeled DRG-Exo by BV2 cells was observed using laser confocal microscopy.

### miRNA microarray analysis

The miRNA microarray analyses of DRG-Exo- and PBS-treated microglia were performed by OE Biotech Company (Shanghai, China). Briefly, total RNA from DRG-Exo- and PBS-treated microglia was extracted, labeled, and processed for hybridization with Agilent SurePrint mouse miRNA microarrays. Microarray data analysis was performed on the Affymetrix miRNA 4.0 platform (Santa Clara, CA, USA).

### Dual-immunofluorescence

The treated BV2 cells were fixed in 4% paraformaldehyde for 0.5 h, and the cells were permeabilized using 0.05% Triton X-100. After sealing with 5% goat serum, the cells were incubated overnight at 4 °C with mouse antibodies to iba1 (1:100, ab283319, Abcam), rabbit antibodies to HECTD1 (1:50, 20605–1-AP, ProteinTech Group, Chicago, IL, USA) and HSP90 (1:50, 4877, Cell Signaling Technologies) and with goat anti-mouse IgG H&L (Alexa Fluor^®^ 647) (1:200, ab150115, Abcam) or goat anti-rabbit IgG H&L (Alexa Fluor^®^ 488) (1:200, ab150077, Abcam) for 60 min at room temperature, respectively. The nuclei were counter-stained with DAPI. The fluorescent staining was observed using laser confocal microscopy.

### Western blot

Mouse spinal cord tissues or BV2 cells were lysed using RIPA lysis buffer containing proteinase inhibitors, and the protein concentration was measured using a BCA protein concentration assay kit. The proteins were separated by SDS-PAGE and transferred to the PVDF membrane. The membranes were sealed in skim milk powder for 45 min and incubated with primary antibodies to iba1 (1:1000, 17198, Cell Signaling Technologies), iNOS (1:1000, 13120, Cell Signaling Technologies), HECTD1 (1:1000, 20605–1-AP, ProteinTech), HSP90 (1:1000, 4877, Cell Signaling Technologies), K63-Ub (1:1000, ab179434, Abcam), and GAPDH (1:2500, ab9485, Abcam) overnight at 4 °C. The proteins were detected by horseradish peroxidase-conjugated goat anti-rabbit IgG H&L (1:2000, ab6721, Abcam) for 2 h at room temperature, and the protein signal was visualized using ECL chemiluminescence. Finally, the grayscale values of the protein bands were measured by Image J.

### Reverse transcription-quantitative polymerase chain reaction (RT-qPCR)

Briefly, the TRIzol kit was used to extract total RNA from mouse spinal cord or BV2 cells, and RNA was reverse transcribed using the PrimeScript RT reagent kit (Perfect Real Time) (RR037Q, Takara Biotechnology Ltd., Dalian, Liaoning, China). For miRNA reverse transcription, miRNA first-strand cDNA synthesis (stem-loop method) was used (B532453-0010, Shanghai Sangon Biological Engineering Technology & Services Co., Ltd., Shanghai, China). Fluorescent qPCR of mRNA or miRNA was performed on an ABI 7900 Fast Real-Time PCR System using TB Green^®^ Premix Ex Taq^™^ II (RR820L, Takara) or miRNA Fluorescent Quantitative PCR Kit (dye method) (B532461-0001, Sangon). Data were normalized to GAPDH or U6, and the relative expression of mRNAs or miRNA was calculated using the 2^−ΔΔCT^ method. All primers used are listed in Table 1.

**Table 1:** Primers used for molecular assays

Gene	Forward sequence (5’–3’)	Reverse sequence (5’–3’)
miR-16-5p	CGCGTAGCAGCACGTAAATA	AGTGCAGGGTCCGAGGTATT
IL-6	TACCACTTCACAAGTCGGAGGC	CTGCAAGTGCATCATCGTTGTTC
IL-1β	TGGACCTTCCAGGATGAGGACA	GTTCATCTCGGAGCCTGTAGTG
HECTD1	GCCTTGATTGGAAGTGGCGAGA	CGGTAAGAGTTTGAGCCACCAG
GAPDH	CATCACTGCCACCCAGAAGACTG	ATGCCAGTGAGCTTCCCGTTCAG
U6	CTCGCTTCGGCAGCACAT	TTTGCGTGTCATCCTTGCG

*miR* microRNA, *HECTD1* HECT domain E3 ubiquitin protein ligase 1

### Enzyme-linked immunosorbent assay (ELISA)

The treated BV2 cells were centrifuged at 1000 g for 15 min at 4 °C. The IL-6 and IL-1β levels in the cell culture supernatant were assessed using the Mouse IL-6 ELISA Kit (ab285330, Abcam) and Mouse IL-1 beta/IL-1F2 Quantikine ELISA Kit (MLB00C, R&D Systems, Minneapolis, MN, USA) according to the manufacturer's protocol.

### Cell scratch assay

The BV2 cells were plated into 6-well plates and cultured until the cell confluence reached 90%. The single cell layer was scratched, and the wound was photographed using a sterilized pipette tip. After washing the cell debris with PBS, the cells were cultured in a serum-free medium for 24 h. The wounds were photographed again, and the wound healing rate was counted at the end of the culture. Wound healing rate = (the wound width_0 h_- the wound width_24 h_)/the wound width_0 h_.

### Co-immunoprecipitation (Co-IP) assay

BV2 cells were lysed in IP lysis buffer containing a mixture of phenylmethanesulfonyl fluoride and proteinase inhibitors according to the protocol of Pierce Classic Magnetic Bead Method IP/Co-IP kit (88804, Thermo Fisher). The supernatant was obtained by centrifugation. Immunoprecipitation reactions were performed using antibodies to HSP90 (1:70, ab203085, Abcam) or IgG (1:100, ab172730, Abcam) overnight at 4 °C to form immune complexes. Protein A/G Magnetic Beads were pre-washed and incubated with the immune complexes for 60 min at room temperature. After the washing of the magnetic beads with IP lysis buffer, the proteins were eluted with elution buffer. Finally, the content of HECTD1 and K63-Ub in the immune complexes was analyzed by western blot.

### Dual-luciferase assay

HECTD1 mRNA 3'-UTR that contained wild-type (WT) or mutant (MUT) miR-16-5p binding sequences were synthesized and cloned into the pGL3 luciferase reporter vector by restriction endosections to generate WT and MUT luciferase reporter vectors. The 293 T cells with miR-16-5p^KD^ were then seeded in 96-well plates and transfected with WT and MUT luciferase reporter vectors. After 48 h of incubation, the Firefly and Renilla luciferase signals were measured using the Dual-Luciferase Assay Kit (Promega Corporation, Madison, WI, USA).

### RNA pull-down assay

For the binding relationship between miR-16-5p and HECTD1 mRNA, we used by MagCapture RNA Pull Down Kit (Lumiprobe Limited, HK, China). After the lysis of 293T cells, the NC-Bio or miR-16-5p-Bio probes (Sangon) were incubated with the lysate for 2 h. The complexes were separated by magnetic beads labeled with streptavidin beads, eluted, and purified. The enrichment of HECTD1 in the enriched products was analyzed by RT-qPCR.

### Data analysis

Experiments were performed in at least three independent biological replicates. Data were expressed as mean ± standard deviation. The results were analyzed using GraphPad Prism 8 software (GraphPad, San Diego, CA, USA). An unpaired *t*-test was used for the statistical analysis of the two groups. For multi-group comparisons, a one-way ANOVA or two-way ANOVA followed by the Student's Newman-Keuls Tukey's or Sidak's post hoc test on the parametric (normality and equal variance pass) data was performed. Significance was set at *p* < 0.05.

## Data Availability

Data sharing does not apply to this article as no datasets were generated or analyzed during the current study.

## Abbreviations

NP: Neuropathic pain
DRG: Dorsal root ganglion
SNL: Spinal nerve ligation
miRNAs: MicroRNAs
HSP90: Heat shock protein 90
HECTD1: HECT domain E3 ubiquitin protein ligase 1
PBS: Phosphate-buffered saline
BCA: Bicinchoninic acid
TEM: Transmission electron microscopy
NTA: Nanoparticle tracking analysis
AAV: Adeno-associated virus
HE: Hematoxylin–eosin
ELISA: Enzyme-linked immunosorbent assay
co-IP: Co-immunoprecipitation
